# A new species of *Telmatobius* (Amphibia, Anura, Telmatobiidae) from the Pacific slopes of the Andes, Peru

**DOI:** 10.3897/zookeys.480.8578

**Published:** 2015-02-02

**Authors:** Alessandro Catenazzi, Víctor Vargas García, Edgar Lehr

**Affiliations:** 1 Department of Zoology, Southern Illinois University, Carbondale, IL 62901, USA; 2 Pro-Fauna Silvestre, Ayacucho, Peru; 3 Department of Biology, Illinois Wesleyan University, P.O. Box 2900, Bloomington, IL 61701, USA

**Keywords:** Huancavelica, amphibian, Andean water frog, Huancavelica, anfibio, rana acuática Andina

## Abstract

We describe a new species of *Telmatobius* from the Pacific slopes of the Andes in central Peru. Specimens were collected at 3900 m elevation near Huaytará, Huancavelica, in the upper drainage of the Pisco river. The new species has a snout–vent length of 52.5 ± 1.1 mm (49.3–55.7 mm, n = 6) in adult females, and 48.5 mm in the single adult male. The new species has bright yellow and orange coloration ventrally and is readily distinguished from all other central Peruvian Andean species of *Telmatobius* but *Telmatobius intermedius* by having vomerine teeth but lacking premaxillary and maxillary teeth, and by its slender body shape and long legs. The new species differs from *Telmatobius intermedius* by its larger size, flatter head, and the absence of cutaneous keratinized spicules (present even in immature females of *Telmatobius intermedius*), and in males by the presence of minute, densely packed nuptial spines on dorsal and medial surfaces of thumbs (large, sparsely packed nuptial spines in *Telmatobius intermedius*). The hyper-arid coastal valleys of Peru generally support low species richness, particularly for groups such as aquatic breeding amphibians. The discovery of a new species in this environment, and along a major highway crossing the Andes, shows that much remains to be done to document amphibian diversity in Peru.

## Abstract (es)

Describimos una nueva especie de *Telmatobius* de la vertiente Pacífica de los Andes en el centro de Perú. Los especímenes tipo fueron colectados a una elevación de 3900 m cerca de Huaytará, Huancavelica, en la parte alta de la cuenca del río Pisco. La nueva especie tiene una longitud hocico–cloaca de 52.5 ± 1.1 mm (49.3–55.7 mm, n = 6) en las hembras adultas y de 48.5 mm en un macho adulto. La nueva especie tiene coloración brillante amarilla y naranja en las partes ventrales y se diferencia fácilmente de todas las demás especies peruanas de *Telmatobius* de los Andes centrales a excepción de *Telmatobius intermedius* por tener dientes vomerianos y carecer de dientes maxilares y premaxilares, y por su forma del cuerpo delgada y sus patas largas. La nueva especie se diferencia de *Telmatobius intermedius* por su mayor tamaño, por tener la cabeza más plana, y por la ausencia de espículas queratinizadas cutáneas (presentes incluso en hembras inmaduras de *Telmatobius intermedius*), y en los machos por la presencia de pequeñas espinas nupciales compactadas en la superficie dorsal y medial de los pulgares (espinas nupciales grandes y dispersas en *Telmatobius intermedius*). Los valles costeros hiper-áridos de Perú se caracterizan en general por tener baja riqueza de especies, especialmente para grupos como los anfibios de reproducción acuática. El descubrimiento de una nueva especie en este tipo de ecosistema, y a lo largo de una de las carreteras principales que cruzan los Andes, muestra que aún queda mucho por hacer para documentar la diversidad de los anfibios en Perú.

## Introduction

The Tropical Andes are characterized by a large diversification of the aquatic frogs of the genus *Telmatobius* Wiegmann, 1834. Sixty-two species are currently recognized in this genus (AmphibiaWeb 2014; Aguilar and Valencia 2009; Frost 2014; including species previously assigned to *Batrachophrynus* Peters, 1873). The altitudinal distribution of *Telmatobius* ranges from 1000 m to 5400 m (De la Riva and Harvey 2003; Seimon et al. 2007), and its longitudinal distribution extends from the equator (*Telmatobius niger* Barbour & Noble, 1920, whose populations have been extirpated in Ecuador; Merino-Viteri et al. 2005) to 29°S, on the eastern slopes of the Argentinean Andes (*Telmatobius contrerasi* Cei, 1977). Twenty-eight species of *Telmatobius* are distributed in Peru (Lehr 2005; AmphibiaWeb 2014), but of these only five [*Telmatobius arequipensis* Vellard, 1955; *Telmatobius intermedius* Vellard, 1955; *Telmatobius jelskii* (Peters, 1873); *Telmatobius peruvianus* Wiegmann, 1834; *Telmatobius rimac* Schmidt, 1954] are known to occur in the hyper-arid coastal valleys that drain directly into the Pacific Ocean.

**Figure 1.**
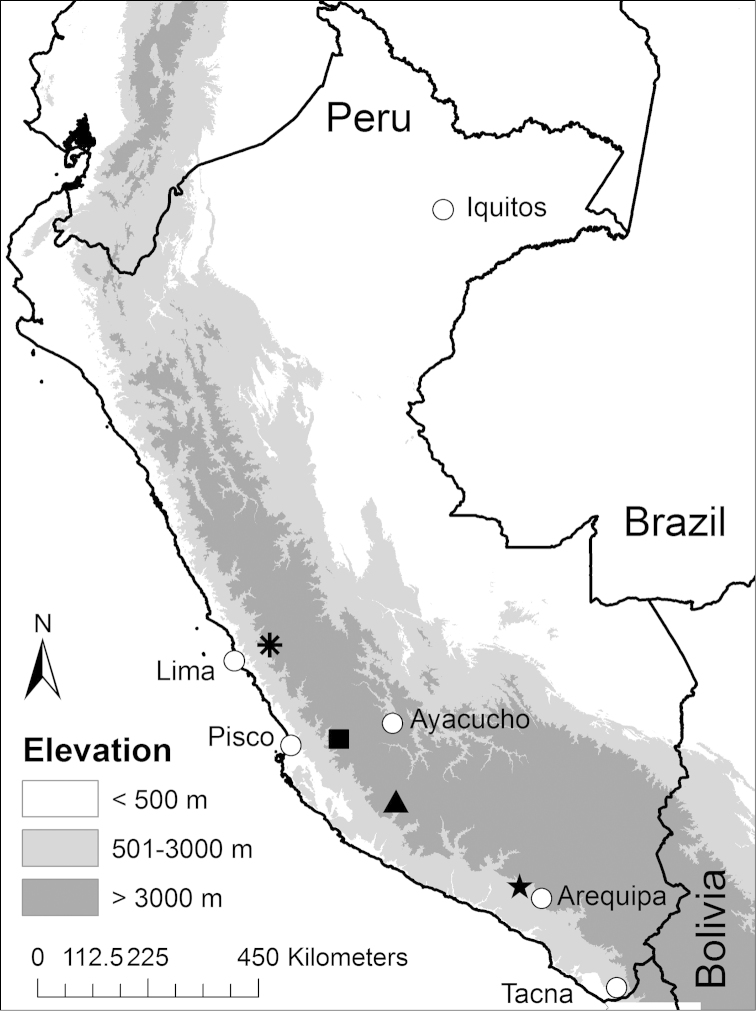
Map of Peru indicating the type localities of *Telmatobius ventriflavum* sp. n. (square), *Telmatobius rimac* (asterisk), *Telmatobius intermedius* (triangle) and *Telmatobius arequipensis* (star). The type locality of *Telmatobius peruvianus* is presumed to be near Tacna.

During October 2012 we made several surveys for the Biodiversity and Monitoring Assessment Program of the Smithsonian Conservation Biology Institute’s Center for Conservation Education and Sustainability (Catenazzi et al. 2013a; Catenazzi et al. 2013b). During one of these surveys, we found a population of *Telmatobius* in the upper drainage of the Huaytará river (Region of Huancavelica), a tributary of the Pisco river in the Pacific slopes of the central Peruvian Andes. Individuals of this population possess traits that do not correspond to the morphological characteristics of other species found in the arid coastal valleys of central Peru (Fig. 1), namely *Telmatobius rimac* to the north and *Telmatobius intermedius* to the south (Vellard 1951; Schmidt 1954; Lehr 2005). Therefore, here we describe the new species and provide a diagnosis to differentiate it from congeneric forms.

## Methods

Specimens were preserved in 10% formol and stored in 70% ethanol. Sex and maturity of specimens were determined externally by observing sexual characters (nuptial spines), and gonads through dissections. Specimens below the SVL of the only male (SVL 48.5 mm) were considered juveniles. We measured the following variables (Table 1) to the nearest 0.1 mm with digital calipers under a stereomicroscope: snout–vent length (SVL), tibia length (TL), foot length (FL, distance from proximal margin of inner metatarsal tubercle to tip of Toe IV), head length (HL, from angle of jaw to tip of snout), head width (HW, at level of angle of jaw), eye diameter (ED), tympanum diameter (TY), interorbital distance (IOD), upper eyelid width (EW), internarial distance (IND), eye–nostril distance (E–N, straight line distance between anterior corner of orbit and posterior margin of external nares). Fingers and toes are numbered preaxially to postaxially from I–IV and I–V respectively. We determined comparative lengths of toes III and V by adpressing both toes against Toe IV; lengths of fingers I and II were determined by adpressing the fingers against each other. Photographs taken by A. Catenazzi were used for descriptions of coloration in life and are available at the Calphoto online database (http://calphotos.berkeley.edu). We used spring scales (Pesola AG, Switzerland) to weigh live specimens in the field. The format of the diagnosis and description follows De la Riva (2005) with the addition of one feature describing head morphology.

**Table 1.** Measurements (in mm) and proportions of type series of *Telmatobius ventriflavum* sp. n.

Characters	Holotype, male	Paratype, female	Paratype, female
	CORBIDI 14685	CORBIDI 14684	CORBIDI 14686
SVL	48.5	52.9	51.5
TL	24.6	27.7	25.5
FL	27.0	31.3	28.7
HL	15.3	16.5	16.0
HW	16.8	17.5	17.0
ED	4.7	4.8	4.7
IOD	3.5	3.9	3.7
EW	3.5	3.6	3.5
IND	3.4	3.5	3.4
E–N	2.7	3.4	3.0
TL/SVL	0.51	0.52	0.50
FL/SVL	0.56	0.59	0.56
HL/SVL	0.32	0.31	0.31
HW/SVL	0.35	0.33	0.33
HW/HL	1.10	1.06	1.06
E–N/ED	0.57	0.71	0.64
EW/IOD	1.00	0.92	0.95

We swabbed specimens in the field to measure infection by *Batrachochytrium dendrobatidis*. Each animal was swabbed with a synthetic dry swab (Medical Wire & Equipment) using a standardized swabbing protocol. In post-metamorphic stages, swabs were stroked across the skin a total of 30 times: 5 strokes on each side of the abdominal midline, 5 strokes on the inner thighs of each hind leg, and 5 strokes on the foot webbing of each hind leg (total of 30 strokes/frog). Tadpoles were swabbed with 10 strokes on the mouthparts. We followed standard DNA extraction and real-time PCR methods (Boyle et al. 2004; Hyatt et al. 2007), except that we analyzed single-swab extracts once instead of three times (Kriger et al. 2006; Vredenburg et al. 2010). We used a real-time Polymerase Chain Reaction (PCR) assay on material collected on swabs to quantify the level of infection (Boyle et al. 2004). This assay compares the sample to a set of standards and calculates a genomic equivalent for each sample, reported as z_e_, the amount of zoospore equivalents (referred as “zoospores” in the text).

Specimens examined are listed in Appendix; codes of collections are: CORBIDI = Herpetology Collection, Centro de Ornitología y Biodiversidad, Lima, Peru; KU = Natural History Museum, University of Kansas, Lawrence, Kansas, USA; MUSM = Museo de Historia Natural Universidad Nacional Mayor de San Marcos, Lima, Peru; USNM = National Museum of Natural History, Washington D.C., USA.

## Taxonomy

### Telmatobius ventriflavum sp. n.

Taxon classificationAnimaliaAnuraTelmatobiidae

http://zoobank.org/E7402169-3339-4218-858A-9CD8DA05A81C

#### Holotype

**Figure 2.**
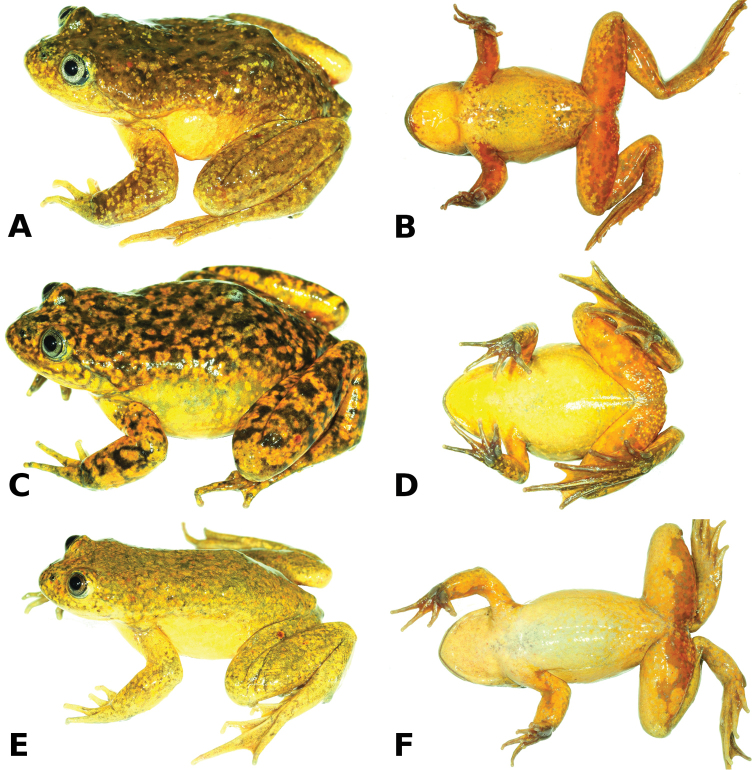
Live holotype of *Telmatobius ventriflavum* sp. n., male CORBIDI 14685 (SVL 48.5 mm) in dorsolateral (**A**) and ventral (**B**) views. Live paratypes, female CORBIDI 14684 (SVL 52.9 mm; **C, D**), and female CORBIDI 14686 (SVL 51.5 mm; **E, F**) in dorsolateral and ventral views. Photographs by A. Catenazzi.

**Figure 3.**
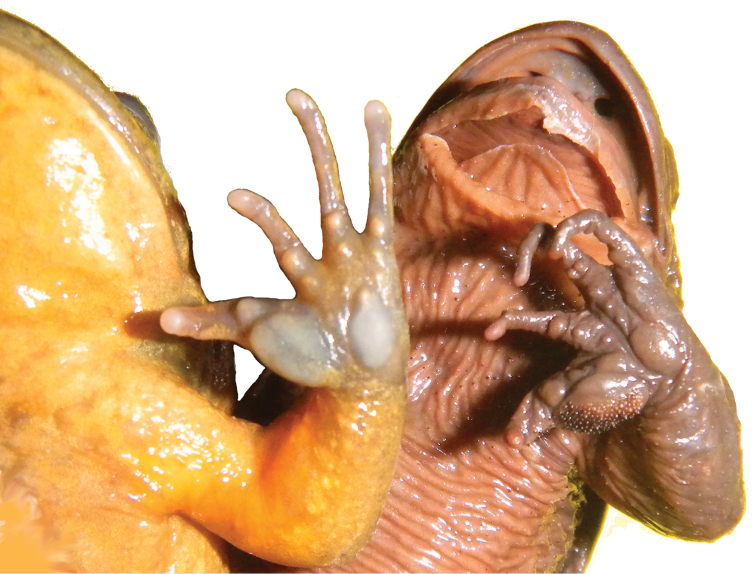
Ventral view of chest and hands showing skin texture on chest and nuptial pads on thumbs in males of *Telmatobius ventriflavum* sp. n. (CORBIDI 14685, *left*) and *Telmatobius intermedius* (MUSM 3752, *right*). Photographs by A. Catenazzi.

**Figure 4.**
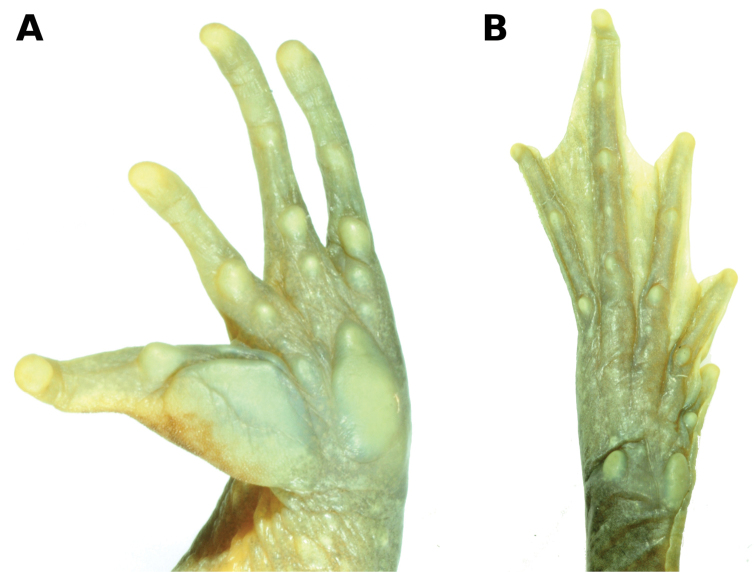
Ventral views of hand (**A**) and foot (**B**) of *Telmatobius ventriflavum* sp. n. (holotype, CORBIDI 14685, SVL 48.5 mm). Photographs by A. Catenazzi.

(Figs 2–4). CORBIDI 14685, an adult male (Figs 2–4) from 13.5806 S, 75.2449 W (WGS84), Chicchobamba, upstream of Represa Negrayccassa, upper drainage of the Huaytará river, 3900 m, Provincia Huaytará, Región Huancavelica, Peru, collected by A. Catenazzi, V. Vargas García, and M. Jaico Huayanay on 18 October 2012.

#### Paratypes

(Fig. 2). Two females (CORBIDI 14684, 14686) collected at the type locality along with the holotype by A. Catenazzi, V. Vargas García and M. Jaico Huayanay on 18 October 2012.

#### Referred specimens.

Six juveniles (MUSM 12748–12750, 12752–12754) and one sub-adult female (MUSM 12755) collected near the type locality (Huaytará; precise location, collector, and date unknown).

#### Diagnosis.

The new species is characterized by (1) snout–vent length of males to 48.5 mm, females to 52.9 mm; (2) head in profile moderately low, with rounded snout; (3) snout rounded in dorsal view; (4) lips not flared; (5) post-commissural gland present, small; (6) tympanum and tympanic annulus not visible; supratympanic fold present; (7) forelimb slender, males lack humeral crest and spine; (8) nuptial spicules minute, closely arranged; nuptial pad on dorsal and medial surface of thumb; no spicules on other fingers; (9) foot fully webbed; palmar and plantar surfaces smooth; (10) tarsal fold present; (11) dorsal skin smooth with small, flat pustules; (12) dorsal surfaces of body and legs golden yellow to golden tan mottled with dark brown, golden yellow and red spots; (13) ventral parts of body yellow, ventral surfaces of limbs marigold or orange; (14) iris light gray with fine black flecks, eye bordered by thin turquoise ring; (15) skull moderately flat, median head length ~78% of head width at the level of the quadratojugal-maxillary articulation, eye-to-nostril distance ~20% of head length.

#### Comparisons.

*Telmatobius ventriflavum* is readily distinguished from all other central Peruvian Andean species (*Telmatobius arequipensis*, *Telmatobius atahualpai*, *Telmatobius brachydactylus*, *Telmatobius brevipes*, *Telmatobius brevirostris*, *Telmatobius culeus*, *Telmatobius jelskii*, *Telmatobius latirostris*, *Telmatobius macrostomus*, *Telmatobius marmoratus*, *Telmatobius mayoloi*, *Telmatobius peruvianus*, *Telmatobius punctatus*, *Telmatobius rimac*, and *Telmatobius truebae*) but *Telmatobius carrillae* and *Telmatobius intermedius* by the absence of premaxillary and maxillary teeth. Furthermore, the species differs from *Telmatobius carrillae* by having vomerine teeth. The new species is readily distinguished from *Telmatobius intermedius* by its larger size reaching 55.7 mm in snout–vent length in females and 48.5 mm in males (45.0 mm in both sexes of *Telmatobius intermedius*), flatter head, the absence of cutaneous keratinized spicules (spicules on dorsal surfaces of skin even in immature females), and in males by the presence of minute, densely packed nuptial spines on dorsal and medial surfaces of thumbs (Fig. 3: large, sparsely packed nuptial spines on dorsal, medial and ventral surfaces). The morphologically similar *Telmatobius peruvianus* is distinguished by having pre-maxillary teeth, skin of dorsum rugose, and by males having large and scattered nuptial spines on thumbs, and keratinized spicules on chest, throat, lower jaw, forearms, hind limbs, and sometimes dorsum. Another species found in coastal river valleys, *Telmatobius rimac* has a much larger body size (to 70.5 mm in males and 86.9 mm in females, Lehr 2005), robust body and limbs, ventral coloration of body brown and of limbs yellow. Males of *Telmatobius arequipensis* have nuptial spines on fingers I and II. The other *Telmatobius* species known to occur in Región Huancavelica, *Telmatobius jelskii* has larger body size (to 68.6 mm in males and 84.7 mm in females), large and conical nuptial spines on thumb and spines on chest and throat in males, and ventral coloration white or light gray, with yellow-orange blotches on hindlimbs.

#### Description of holotype.

An adult male with a SVL of 48.5 mm; body slender; head slightly wider than long, its length 31.5% of SVL; head width 34.6% of SVL; head length 91.1% of head width. Head flat; snout rounded in lateral and dorsal views; nostrils not protuberant, oriented dorsally; internarial distance 20.2% of head width; nostrils closer to margin of orbit than to tip of snout; internarial region slightly convex; eye large, 30.7% of head length; loreal region moderately concave; lips not flared; tympanum and tympanic annulus indistinct; supratympanic fold well developed, extending from behind eye to level of shoulder; distinct dermal fold from supratympanic fold to post-commissural gland; post-commissural gland small, oval. Maxillary and premaxillary teeth absent; dentigerous processes of vomers between choanae, each bearing two small, fanglike teeth; choana width 1.6 mm, subcircular; tongue rounded, attached anteriorly through about one third of its length; vocal slits absent.

Forelimb slender; humeral crest absent (Fig. 4A); relative lengths of fingers: I = II < III > IV (Fig. 4A); Finger II notably shorter than Finger IV; palmar webbing absent; tips of fingers spherical; fingers lacking lateral fringes; inner palmar (prepollical) tubercle oval, distinct; outer palmar tubercle slightly larger than inner, oval; subarticular tubercles rounded; five or six rounded supernumerary palmar tubercles, palmar surface smooth; nuptial excrescence on medial surface of thumb, composed by minute, keratinized, brown spicules closely arranged, forming a pad in contact with inner palmar tubercle posteriorly; keratinized spicules not covering surfaces of Finger II. Hind limbs long and slender; tibia length 50.7% of SVL; foot length 55.7% of SVL, combined lengths of tibia and foot 106.4% SVL; upper surfaces of hind limbs smooth; relative lengths of toes: I < II < III < IV > V (Fig. 4B); Toe V slightly longer than Toe III; toes fully webbed; tips of toes spherical, about the same size of those of fingers; inner metatarsal tubercle moderately flattened, oval; outer metatarsal tubercle round, about two-thirds the size of inner metatarsal tubercle; subarticular tubercles round or slightly oval; four supernumerary tubercles small and rounded, plantar surface smooth.

Skin on dorsum smooth with small, flat pustules; keratinized spicules or spines absent; loose folds of skin absent; ventral skin smooth; cloacal opening approximately at dorsal level of thighs.

Measurements of holotype provided in Table 1.

#### Coloration of holotype in alcohol.

Dorsal surfaces of head, body, and limbs grayish-brown, with dark spots discernible on dorsum; spots that are golden yellow and red in life discernible as light grey spots; throat and venter light yellowish-gray; ventral surfaces of limbs tan yellow with yellowish-gray spots; plantar and palmar surfaces gray; tips of digits cream.

#### Coloration of holotype in life.

Dorsal surfaces golden tan mottled with dark brown, golden yellow and red spots especially on scapular region and on head; flanks tan yellow; throat and venter golden yellow to marigold, fading into depigmented spots on chest; ventral surfaces of limbs at points of insertion quickly transitioning from tan yellow or marigold to orange distally and posteriorly; iris light gray with fine black flecks; eyes bordered by a thin turquoise ring.

#### Variation.

Coloration in life is based on field notes and photographs taken by A. Catenazzi (Fig. 2) of six females (the two paratypes and four uncollected females) and one male (the holotype) found at the type locality. The only captured male is smaller than females (Table 1) and weighted 13.6 g. The SVL of females averaged 52.5 ± 1.1 mm (49.3–55.7 mm), whereas their weights averaged 16.2 ± 1.3 g (11.5–19.7 g). A juvenile with 43.4 mm in SVL weighed 8.4 g. Some individuals (e.g., paratype CORBIDI 14686, Fig. 2E) had much lighter, predominantly golden yellow dorsal coloration than the holotype, whereas most individuals (e.g., paratype CORBIDI 14684, Fig. 2C) had dorsal mottling similar or darker than the coloration of the holotype. The ventral coloration seemed to vary with sex. Whereas the male had extensive and bright marigold to orange coloration on the ventral parts of the limbs, in females such coloration was more suffused, and much of the ventral parts of the limbs presented the same yellow coloration of venter and throat. In all captured individuals the irises were light grey and the eyes were surrounded by a thin turquoise ring.

#### Etymology.

The specific name *ventriflavum* is derived from Latin nouns *venter*, meaning belly, and *flavus*, meaning yellow. The species epithet refers to the golden yellow and orange coloration on the ventral parts of the body and limbs.

#### Distribution, natural history, and threats.

**Figure 5.**
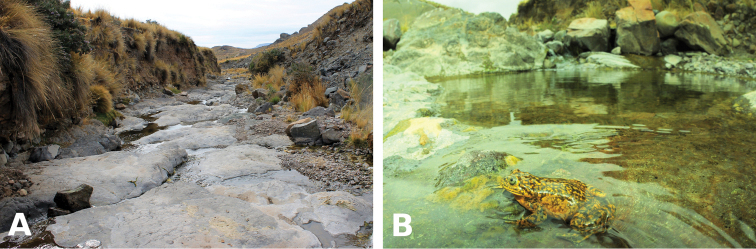
Habitat of *Telmatobius ventriflavum* at the type locality (**A**) and detail of stream pool used by adults and tadpoles (**B**). Photographs by V. Vargas García (**A**) and A. Catenazzi (**B**).

The new species is only known from the type locality (Fig. 5), a stream tributary of the Huaytará river, which is in the upper drainage of the Pisco river watershed. The Pisco watershed drains directly into the Pacific Ocean, and is dominated by arid and hyper-arid environments, especially at elevations below 3000 m. The habitat surrounding the type locality at 3900 m is sparse puna. Rainfall is concentrated during the months from January to March, with very little precipitation occurring during the remaining months. The type locality is a stream ~10 m wide, with a rocky substrate that alternates pebble and gravel pools with small waterfalls and riffles. However, 500 m downstream of the type locality, the stream has been dammed to build a reservoir. The habitat downstream of the reservoir is not suitable to *Telmatobius*, although the reach was not inspected during our visit. The reach upstream of the type locality has not been surveyed and extends up to 4300 m in elevation. This reach appears to be in good condition and could be used by the species for reproduction.

The valley of the type locality and adjacent central Peruvian coastal valleys are dominated by arid environments that likely limit dispersal of the species. Therefore, and considering that other species (*Telmatobius rimac* to the north, and *Telmatobius intermedius* to the south) are known from nearby coastal regions, it is very likely that the new species is endemic to the upper Pisco watershed and adjacent river basins. It is unknown whether the species overlaps geographically with *Telmatobius rimac* or *Telmatobius intermedius*, or with the highland species *Telmatobius jelskii* which occurs in the adjacent Región Ayacucho. During the visit on 18 October 2012, three observers searched 100 m of the stream reach during 60 minutes (search effort of 180 person-minutes), and encountered 43 tadpoles, one juvenile, 6 females and one male. The tadpoles were in developmental stages 25 to 45 (following Gosner 1960), suggesting that the species is breeding successfully over extended periods of time including during the dry season (October is at the end of the dry season when stream levels are low).

We detected the presence of the pathogenic fungus *Batrachochytrium dendrobatidis* Longcore, Pessier & Nichols, 1999 at the type locality. The prevalence of infection during our visit on 18 October 2012 was 53.5% for tadpoles (n = 43) and 40.0% for adults (n = 5); the only juvenile found was infected. Among tadpoles, prevalence was 23.1% for larval stages (Gosner stages 25 to 39, n = 13), and 66.7% for pre-metamorphic stages (Gosner stages 40 to 45, n = 30). Among infected individuals, infection loads were very small for adults and the juvenile (0 < z_e_ < 1 zoospores), and ranged from 1.6 to 2162.4 zoospores for tadpoles (average z_e_ = 177.3 ± 66.4 zoospores; median = 76.1 zoospores).

## Discussion

Species of *Telmatobius* are known to exhibit considerable intraspecific variation in morphological and meristic traits, which hinders the use of these traits in taxonomy and systematics (Aguilar and Valencia 2009; De la Riva et al. 2010; Sáez et al. 2014). However, the unique combination of traits and coloration of *Telmatobius ventriflavum* warrants its description as a separate species. The new species bears some similarity with *Telmatobius intermedius* and *Telmatobius peruvianus*. The three species are medium sized, slender with relatively long tibias and feet, head wider than longer and short snout. They also inhabit similar habitats, high-elevation rocky streams that are tributary of coastal rivers draining directly into the Pacific Ocean. However, the new species is larger (the only known male is larger than the largest females of *Telmatobius intermedius* and *Telmatobius peruvianus*), both sexes have smooth skin, and males have small and densely packed nuptial spines.

Admittedly our comparisons of adult specimens are not ideal. For example, only one specimen of *Telmatobius intermedius* was available for comparison. However, this single specimen was a male exhibiting the large nuptial spine and the dorsal skin covered by keratinized spicules (Fig. 3). Concerning *Telmatobius peruvianus*, the original type locality of “Peru” (Wiegmann 1834) had been restricted to the Cordillera de Guatilla in Chile (Schmidt 1928), with several subsequent authors referring to Chilean material for morphological descriptions. The taxonomic status of the Chilean populations of *Telmatobius peruvianus* has recently been questioned (Sáez et al. 2014), and these populations might belong to a different taxon than the Peruvian populations. However, for our present work we only relied on specimen descriptions (Vellard 1951) and direct comparison of material collected near Tacna, Peru (Appendix).

A comparison of the larval stages of these three species could add additional diagnostic characters, but the tadpole of *Telmatobius intermedius* has not been collected and the tadpole of *Telmatobius peruvianus* has been described from specimens collected in Chile. We postpone description of the tadpoles of *Telmatobius ventriflavum* until tadpoles of *Telmatobius intermedius* and of *Telmatobius peruvianus* from Peruvian populations become available. Further research is needed to establish the phylogenetic relationships of these three taxa. Recent research from Chile revealed that species inhabiting the western valleys of the Andes formed an endemic lineage that diverged from lineages of the Altiplano in the late Pleistocene (Sáez et al. 2014).

The discovery of a new species in the species-poor coastal valleys of central Peru is unusual because these areas have been surveyed before and are easily accessible. For example the stream at the type locality of *Telmatobius ventriflavum* is at the crossing with the highway connecting the coastal Panamerican highway to the city of Ayacucho. Furthermore, two other species of *Telmatobius* were already known from coastal valleys to both the north (~200 km, *Telmatobius rimac*) and the south (~170 km, *Telmatobius intermedius*) of the type locality of *Telmatobius ventriflavum*. In such arid landscape one would not expect to find several species of aquatic frogs. Specimens of the new species had previously been collected from Huaytará, and had been deposited at the MUSM as unidentified *Telmatobius*. These specimens were not included in the type series because they are not adults, and because the name of collectors, and date and precise location of collection are unknown. Nevertheless, the discovery of a new species in such arid and easily accessible environments shows that much remains to be done to document amphibian diversity in the Andes (Sáez et al. 2014).

In addition to probably having a restricted geographic distribution, *Telmatobius ventriflavum* is threatened by the presence at the type locality of the pathogenic fungus *Batrachochytrium dendrobatidis*. This fungus causes chytridiomycosis and has been implicated in population declines and species extinctions of species of *Telmatobius* throughout the Andes (Merino-Viteri et al. 2005; Barrionuevo and Mangione 2006; Seimon et al. 2007; Barrionuevo and Ponssa 2008; Catenazzi et al. 2011; De la Riva and Burrowes 2011; Catenazzi et al. 2014; Catenazzi and von May 2014). For example, the three *Telmatobius* species known from Ecuador were extirpated in the late 1980s and early 1990s and are now thought to be extinct (Merino-Viteri et al. 2005). The last individuals of *Telmatobius* found in Ecuador showed symptoms of chytridiomycosis (Merino-Viteri et al. 2005). Moreover, population declines of *Telmatobius marmoratus* and *Telmatobius timens* in Peru (Seimon et al. 2007; von May et al. 2008; Catenazzi et al. 2011) and of three species of *Telmatobius* in Argentina (Barrionuevo and Ponssa 2008) have been associated with outbreaks of *Batrachochytrium dendrobatidis*, and high prevalence of infection has been reported from high-elevation populations of *Telmatobius jelskii* in central Peru (Catenazzi et al. 2013b) and *Telmatobius gigas* in Bolivia (De la Riva and Burrowes 2011). Therefore, further studies should monitor infection and assess the susceptibility of *Telmatobius ventriflavum* to chytridiomycosis. Additional threats to the conservation of the species are the damming and regulation of streams, large-scale infrastructure projects (reservoirs, pipelines, roads, etc.) and contamination from mining and agriculture.

## Supplementary Material

XML Treatment for Telmatobius ventriflavum

## Appendix

**Specimens examined**

*Telmatobius arequipensis* (5 specimens): PERU: Moquegua: Mariscal Nieto, Qda. Torota, MUSM 19491, 19493, 19474, 19492, 19494.

*Telmatobius intermedius* (1 specimen): PERU: Ayacucho: near Puquio, Allipaca, MUSM 3752 (lectotype).

*Telmatobius jelskii* (6 specimens): PERU: Ayacucho: Tambo, La Mar, MUSM 7646 (lectotype); Ayacucho: Seccelambra, MUSM 28511; Abra Toccto, MUSM 28513–14, Vinchos, MUM 28517; Huancavelica: Huancavelica, MUSM 7639 (lectotype).

*Telmatobius mayoloi* (5 specimens): PERU: Ancash: Conococha, MUSM 20470, 20742–74, 20489.

*Telmatobius peruvianus* (9 specimens): PERU: Tacna: Caplina, MUSM 19604–6, 19608; Putre, FMNH 6385, 6390, 6394–96, 6399–6400.

*Telmatobius rimac* (17 specimens): PERU: Lima: 6 km WSW Matucana, KU 181474–84; Canta, MUSM 19640–41, 19643, 19646, 19648; Tupe, MUSM 7657.
